# Facial Diplegia Variant of Guillain-Barré Syndrome in Pregnancy Following COVID-19 Vaccination: A Case Report

**DOI:** 10.7759/cureus.22341

**Published:** 2022-02-17

**Authors:** Adeel S Zubair, Ju Young Bae, Kunal Desai

**Affiliations:** 1 Neurology, Yale School of Medicine, New Haven, USA; 2 Department of Internal Medicine, Yale New Haven Health Greenwich Hospital, Greenwich, USA

**Keywords:** guillain-barré syndrome (gbs), neuromuscular, facial diplegia, covid-19 vaccination, covid-19

## Abstract

Serious neurologic complications from coronavirus disease 2019 (COVID-19) vaccination are rare, and only a few cases of Guillain-Barré syndrome (GBS) have been reported after COVID-19 vaccination. We present the first reported case of the facial diplegia variant of GBS after recent COVID-19 vaccination in a pregnant woman. The 30-year-old patient was 27 weeks pregnant at the time she was diagnosed with the facial diplegia variant of GBS. Her symptoms began two weeks after she received the Ad26.COV2.S COVID-19 vaccine. A thorough evaluation for GBS was done, including a lumbar puncture that demonstrated elevated cerebrospinal fluid (CSF) protein and nerve conduction study (NCS) that found evidence of a diffuse sensorimotor demyelinating polyneuropathy. Nasal swab testing for severe acute respiratory syndrome coronavirus 2 (SARS-CoV-2) was negative on two occasions five days apart. All other diagnostic testing was unremarkable or nonexplanatory of the patient’s clinical presentation. She was started on intravenous immunoglobulin (IVIG) and had significantly improved dysphasia, dysarthria, and facial strength. The patient recovered to baseline four weeks after presentation.

## Introduction

Serious neurologic complications from coronavirus disease 2019 (COVID-19) vaccination are rare, and only a few cases of Guillain-Barré syndrome (GBS) have been reported after COVID-19 vaccination. GBS is a neuromuscular disorder comprising of a group of acute immune-mediated disorders that affect the peripheral nerves by either attacking the axons or myelin [1]. It is often postinfectious but has been described postvaccination or with no identifiable cause [1]. The facial diplegia variant of GBS is rare and can lead to significant morbidity and mortality, and there is one reported case of this variant potentially related to recent COVID-19 [2]. The facial diplegia variant of GBS often presents with facial weakness, often involving both sides. It is estimated to occur in less than 1% of patients diagnosed with GBS [3]. There are a variety of conditions that can also lead to bilateral facial diplegia, including leprosy, Bell’s palsy, Lyme disease, sarcoidosis, and brainstem stroke, among others [3]. This can result in difficulty arriving at a diagnosis, and providers need to be on the lookout for this variant as a cause as well. In an era of ever-increasing COVID-19 variants and significant vaccine hesitancy, transparency in discussing potential concerns with vaccines is key to maintaining public trust. We present the first reported case of the facial diplegia variant of GBS in a pregnant woman after receiving a COVID-19 vaccine. An extensive diagnostic workup was performed, and the patient responded very well to treatment. We also review recent data in favor of vaccination despite concerns about GBS.

## Case presentation

A 30-year-old, right-handed, pregnant gravida 1 parity 0 (G1P0) female with an estimated gestational age of 27 weeks presented to the emergency department (ED) with four days of bifrontal headache, new-onset left facial weakness, and loss of taste. Her past medical history was notable for heterozygous factor V Leiden deficiency, not currently on treatment. She had weakness of the upper and lower face, and both hyperacusis and dysgeusia on the left. The patient denied any antecedent infections, tick bites, surgical procedures involving her face or mouth, or health changes. To date, she had no obstetric issues. She had received the Ad26.COV2.S COVID-19 vaccine two weeks prior to symptom onset. Severe acute respiratory syndrome coronavirus 2 (SARS-CoV-2) polymerase chain reaction (PCR), complete blood count (CBC) with differential, complete metabolic panel (CMP), Lyme antibodies, erythrocyte sedimentation rate (ESR), C-reactive protein (CRP), hemoglobin A1c, and vitamin B12 were unremarkable. Magnetic resonance imaging (MRI) of the brain demonstrated craniovertebral junction tonsillar extension into the foramen magnum by 4-5 mm, consistent with a borderline Chiari 1 malformation but no other abnormalities. Head magnetic resonance venography (MRV) found no evidence of acute dural venous sinus thrombosis. She was discharged on prednisone taper and valacyclovir with a diagnosis of facial nerve palsy and instructed to pursue outpatient neurology follow-up.

Five days after the initial presentation, she developed facial diplegia with dysarthria, dysphagia, and hand paresthesia. She returned to the ED and was tachycardic, normotensive, and afebrile. Neurologic examination was notable for normal mental status, equal and reactive pupils, intact extraocular movements, decreased facial sensation, facial diplegia, and impaired taste. Her speech was dysarthric. Motor examination included normal strength and tone in the upper and lower extremities with sensory examination notable for decreased pinprick sensation in the hands and feet. Reflexes were present and symmetric throughout with down-going toes. She had a normal gait with negative Romberg. Cerebrospinal fluid (CSF) analysis was notable for zero nucleated cells, elevated protein count of 104.3 mg/dL, and glucose of 59 mg/dL. Other extensive workup was completed with the results summarized in Table 1, Table 2, and Table 3. Reverse transcription-polymerase chain reaction (RT-PCR) for SARS-CoV-2 was negative on two occasions five days apart. Similarly, serum Lyme antibody testing and antinuclear antibody (ANA) testing were both negative on two occasions five days apart.

**Table 1:** Serum workup CMV: cytomegalovirus

Laboratory data	Reference range	Value
Autoimmune		
Antinuclear antibody (ANA)	<1:80	1:40
Angiotensin-converting enzyme	12–82 U/L	17 U/L
Acetylcholine receptor (muscle) binding antibody	≤0.02 nmol/L	0 nmol/L
Ganglioside ASIALO GM 1 antibody, IgG, enzyme immunoassay (EIA)	<1:100 titer	1:100 (H)
Ganglioside ASIALO GM 1 antibody, IgM, EIA	<1:1600
Ganglioside GD1A antibody, IgG, EIA	<1:100 titer	<1:100
Ganglioside GD1A antibody, IgM, EIA	<1:800 titer	<1:800
Ganglioside GQ1B antibody, IgG	<1:100 titer	<1:100
GM 1 IgG	<1:800 titer	<1:800
GM 1 IgM	<1:800 titer	<1:800
Hematologic/endocrinologic		
Vitamin B12	211–911 pg/mL	412 pg/mL
Methylmalonic acid	0.00–0.40 umol/L	0.1 umol/L
Vitamin B1	8–30 nmol/L	18 nmol/L
Vitamin B3 (niacin)	<20 ng/mL (fasting)	<20 ng/mL
Nicotinamide	<40 ng/mL (fasting)	33 ng/mL
Vitamin B6	2.1–21.7 ng/mL	15.5 ng/mL
Total protein, serum	6–8.3 g/dL	5.8 g/dL (L)
Albumin, serum	3.6–4.9 g/dL	3.8 g/dL
Alpha-1 globulin	0.10–0.30 g/dL	0.24 g/dL
Alpha-2 globulin	0.60–1 g/dL	0.84 g/dL
Gamma globulin	0.70–1.50 g/dL	0.60 g/dL (L)
Serum protein electrophoresis (physician Interpretation)	No discrete abnormal bands	No discrete abnormal bands
Immunoglobulin A	70–470 mg/dL	113 mg/dL
Immunoglobulin M	40–230 mg/dL	101 mg/dL
Immunoglobulin G	700–1,600 mg/dL	754 mg/dL
Immunofixation electrophoresis (physician Interpretation)	No definite bands are identified	No definite bands are identified
Thyroid-stimulating hormone	0.320–3.850 µIU/mL	2.830 µIU/mL
Hemoglobin A1c	<5.7%	4.9%
Infectious		
Human immunodeficiency virus PCR	Not detected	Not detected
Hepatitis B surface antigen	Negative	Negative
Hepatitis B surface antibody	≥12 mIU/mL	128.82 mIU/mL
Hepatitis C antibody with reflex to HCV PCR	Negative	Negative
Total treponema pallidum antibody, serum	Nonreactive	Nonreactive
CMV IgG	Negative	Positive (A)
CMV IgM	<8.00 AU/mL	Negative
Lyme antibody	≤0.90 LI	0.39 LI

**Table 2:** Urine testing

Urinalysis	Reference range	Value
Clarity	Clear	Clear
Color	Yellow	Yellow
Specific gravity	1.005–1.030	1.011
pH	5.5– 7.5	7.0
Protein	Negative (trace)	Negative
Glucose	Negative	Negative
Ketones	Negative	Negative
Blood	Negative	Negative
Bilirubin	Negative	Negative
Leukocytes	Negative	Negative
Nitrite	Negative	Negative
Urobilinogen	≤2 EU/dL	<2 EU/dL
Nine drug toxicology panel		
Amphetamine	Negative	Negative
Benzodiazepines	Negative	Negative
Cannabinoids	Negative	Negative
Cocaine	Negative	Negative
Opiates	Negative	Negative
Oxycodone	Negative	Negative
Phencyclidine	Negative	Negative
Methadone metabolite	Negative	Negative
Barbiturate	Negative	Negative
Urine protein electrophoresis		
Albumin	Not established	0.01 g/L
Alpha-1 globulin	Not established	0.00 g/L
Alpha-2 globulin	Not established	0.01 g/L
Beta globulin	Not established	0.01 g/L
Gamma globulin	Not established	0.02 g/L
Urine protein electrophoresis (physician Interpretation)	No abnormal proteins	No abnormal proteins

**Table 3:** Cerebrospinal fluid testing

Cerebrospinal fluid (CSF)	Reference range	Value
Nucleated cell count, tube #1	<6 cells/uL	0 cells
Red cell count, tube #1	None	1 cell
Glucose, CSF	40–70 mg/dL	59 mg/dL
Protein, total CSF	15–45 mg/dL	104.3 mg/dL
Herpes simplex virus by polymerase chain reaction (PCR)	Not detected	Not detected
Varicella zoster PCR	Not detected	Not detected
Epstein–Barr virus by PCR	Not detected	Not detected
Fungal culture	No growth after four weeks	No growth after four weeks

The patient was diagnosed with the facial diplegia variant of GBS and was started on intravenous immunoglobulin (IVIG) at 2 g/kg after pretreatment with acetaminophen and diphenhydramine. She was monitored closely with testing for negative inspiratory force and vital capacity as well as regular fetal assessments by obstetrics service. She also received eye drops to treat dry eyes and was cleared for diet after speech and swallow evaluation. The patient had significantly improved dysphasia, dysarthria, and facial strength with IVIG. Additionally, her numbness began resolving, and her voice began returning to normal. Once she reached 28 weeks estimated gestational age, she received a RhoGAM shot with no complications. The patient fully recovered to baseline four weeks after presentation. Once she reached 40 weeks and five days gestational age, the patient gave birth to a healthy baby boy via normal spontaneous vaginal delivery with no complications.

## Discussion

Our patient had no reported history of infection or signs of ongoing infection at the time of presentation. Workup identified positive cytomegalovirus (CMV) IgG and negative CMV IgM consistent with prior CMV infection that would not explain her symptoms. The findings of facial diplegia occurring in a progressive manner with associated sensory symptoms coupled with the albuminocytologic dissociation in the CSF and electrodiagnostic findings are consistent with the facial diplegia variant of GBS.

Diagnosis of this rare variant of GBS can be difficult. It is important for providers to keep a broad differential and consider all potential options, including brainstem stroke, sarcoidosis, infectious causes, and Bell’s palsy [3]. As done in this case, further workup including CSF analysis and EMG/nerve conduction study (NCS) helped obtain confirmatory data regarding this diagnosis. The pathophysiology for this condition is not fully elucidated at this time, and further research is ongoing.

The three COVID-19 vaccines approved by the Food and Drug Administration for use are produced by Pfizer-BioNTech (BNT162b2), Moderna (mRNA-1273), and Johnson and Johnson (Ad26.COV2.S). Other countries use vaccines made by AstraZeneca (ChAdOx1-S) or Sinovac (CoronaVac). Our patient had received the Ad26.COV2.S vaccine, which is adenovirus vector-mediated, two weeks prior to the onset of symptoms. There are two reported cases of GBS in the Ad26.COV2.S vaccine trial, one in the treatment arm and one in the placebo arm [4]. GBS has also been reported in patients receiving other forms of COVID-19 vaccine (Table 4) [5-14]. Out of a sample of 15 cases including ours, 10 were men compared to five women. Four of the 15 cases developed GBS after the second dose of a vaccine, compared to the remaining 11 who developed symptoms after one dose. Age at diagnosis ranged from 20 to 82 years old, with a mean age of 55.7 years old. Days from vaccination to GBS symptom onset ranged from four to 40 days, with a mean of 16.7 days, although one case may have been confounded by acute COVID-19 that developed after vaccination and before GBS symptom onset.

**Table 4:** Summary of published cases of Guillain-Barré syndrome after COVID-19 vaccination Pfizer-BioNTech: BNT162b2; Moderna: mRNA-1273; Johnson and Johnson: Ad26.COV2.S; AstraZeneca: ChAdOx1-S; Sinovac: CoronaVac

Case	Age	Gender	Vaccine	Dose	Days from vaccine to onset	Acute COVID-19 after vaccine	Reference
1	60	Female	Ad26.COV2.S	1	10	No	[4]
2	37	Male	ChAdOx1	1	14	No	[5]
3	82	Female	BNT162b2	1	14	No	[6]
4	48	Male	ChAdOx1	1	10	No	[7]
5	73	Male	BNT162b2	2	20	No	[8]
6	32	Male	Unknown	1	8	No	[9]
7	54	Male	ChAdOx1	1	16	No	[10]
8	20	Male	ChAdOx1	1	26	No	[11]
9	57	Male	ChAdOx1	1	21	No	[12]
10	55	Male	ChAdOx1	1	29	No	[13]
11	61	Male	mRNA-1273	2	4	No	[14]
12	69	Female	ChAdOx1-S	1	40	Yes	[15]
13	82	Female	BNT162b2	2	14	No	[16]
14	76	Male	CoronaVac	2	8	No	[17]
15	30	Female	Ad26.COV2.S	1	17	No	Our case

A US-based population study of pregnant women aged 10-55 calculated the incidence of GBS during pregnancy as 2.8 per million person-years (95% CI: 0.5%-9.3%) [15]. The incidence of GBS in the general population is 0.16-3 per 100,000 person-years [16]. To our knowledge, this is the first reported case of the facial diplegia variant GBS after COVID-19 vaccination in a woman who is pregnant. Although there is no thorough safety data available on COVID-19 vaccine and pregnancy, the Centers for Disease Control and Prevention and the American College of Obstetricians and Gynecologists support COVID-19 vaccination in pregnancy [17].

## Conclusions

While this patient presented after COVID-19 vaccination without preceding infection or other treatment linked with GBS, that does not by itself implicate the vaccine as a cause. We stress that temporal associations do not imply causality. Rather, we highlight this case to encourage additional large population-based studies to assess for any statistically significant correlations. COVID-19 vaccine is critically important in turning the tide against this pandemic, and we strongly recommend vaccination given the significant benefits.
